# *Crocus sativus* L. Extracts and Its Constituents Crocins and Safranal; Potential Candidates for Schizophrenia Treatment?

**DOI:** 10.3390/molecules26051237

**Published:** 2021-02-25

**Authors:** Nikolaos Pitsikas

**Affiliations:** Department of Pharmacology, School of Medicine, Faculty of Health Sciences, University of Thessaly, Biopolis, Panepistimiou 3, 415-00 Larissa, Greece; npitsikas@med.uth.gr; Tel.: +30-2410-685-535

**Keywords:** *Crocus sativus* L., crocins, schizophrenia

## Abstract

Schizophrenia is a chronic mental devastating disease. Current therapy suffers from various limitations including low efficacy and serious side effects. Thus, there is an urgent necessity to develop new antipsychotics with higher efficacy and safety. The dried stigma of the plant *Crocus sativus* L., (*CS*) commonly known as saffron, are used in traditional medicine for various purposes. It has been demonstrated that saffron and its bioactive components crocins and safranal exert a beneficial action in different pathologies of the central nervous system such as anxiety, depression, epilepsy and memory problems. Recently, their role as potential antipsychotic agents is under investigation. In the present review, I intended to critically assess advances in research of these molecules for the treatment of schizophrenia, comment on their advantages over currently used neuroleptics as well-remaining challenges. Up to our days, few preclinical studies have been conducted to this end. In spite of it, results are encouraging and strongly corroborate that additional research is mandatory aiming to definitively establish a role for saffron and its bioactive components for the treatment of schizophrenia.

## 1. Schizophrenia

Schizophrenia is a serious chronic mental disease that affects up to 1% of the world population. It is a complex heterogeneous psychiatric disorder that impairs social, occupational and individual functioning and causes an adjective decrease in the quality of life of patients. This disease usually is manifested in late adolescence or early adulthood. Schizophrenics display serious psychotic symptoms, which can be classified into three major categories: positive symptoms (e.g., hallucinations, delusions, disordered thinking, catatonic behavior), negative symptoms (e.g., social withdrawal, anhedonia, avolition, neglect of hygiene) and cognitive disturbances (e.g., in attention, executive functioning and memory) [1].

Schizophrenia’s causes and pathophysiology are not yet elucidated. Nevertheless, it is widely acknowledged as a composite neurodevelopmental disease affected by genetic and environmental factors [2,3]. Specifically, it has been revealed that monozygotic siblings of schizophrenics have a 50–80% risk of developing the disease. Further, incomplete maturation of the brain and abnormal synaptic connections between different brain areas are also evidenced [4] Interestingly, an increasing number of reports propose the implication of oxidative stress in the pathophysiology of schizophrenia [5].

Additionally, several lines of evidence suggest that malfunctioning of different neurotransmitter systems, as are dopamine (DA), glutamate, cholinergic, serotonergic and the GABAergic systems is associated with the appearance of this disease [6]. In particular, positive symptoms of schizophrenia are associated with overactivation of dopaminergic neurotransmission in the striatum, while negative symptoms and cognitive impairments appear to be dependent on dopaminergic hypofunction in the prefrontal cortex [7].

Glutamate hypofunction seems also to be involved in the pathophysiology of schizophrenia. Abnormal glutamatergic transmission is related to secondary dopaminergic dysfunction in the striatum and prefrontal cortex. In this context, it has been shown that pharmacological blockade of NMDA receptor induces negative symptoms and cognitive deficits that were not alleviated by neuroleptics [7]. Moreover, the functionality of the GABAergic system, the major inhibitory neurotransmitter in the brain, is compromised in schizophrenia [8]. Since GABAergic firing modulates dopaminergic transmission in the prefrontal cortex, the malfunctioning of GABA interneurons seems to play a role in the appearance of some of the clinical symptoms of schizophrenia [9].

Clinical findings indicate that conventional antipsychotics (either those of the first generation, or atypical) display a certain efficacy in the alleviation of positive symptoms but are inefficacious in relieving negative symptoms and cognitive impairments of schizophrenia patients. These medications, however, are associated with important side effects which compromise their benefit. Specifically, motor side effects (Parkinsonism) are related with the administration of traditional neuroleptics (e.g., chlorpromazine, haloperidol). Conversely, the administration of atypical antipsychotics (e.g., clozapine, olanzapine, risperidone) does not produce Parkinsonism but causes weight gain. In addition, 30% of patients are resistant to the above-described treatments. Collectively, these results suggest that there is a pressing necessity to find novel compounds which could provide alleviation of negative symptoms and cognitive deficits typical features of schizophrenia patients [10,11].

Among the different alternative approaches for the therapy of schizophrenia, the involvement of the plant saffron and its bioactive components as potential anti-schizophrenia agents has lately been suggested. In the current analysis, I intend to assess with critical feeling the potential beneficial action of saffron and its components for the treatment of schizophrenia.

## 2. *Crocus sativus* L. (Saffron)

*Crocus sativus* L. (*CS*), is a perennial herb and a member of the Iridaceae family, of genus *Crocus*, the line of Liliaceae. This plant is cultivated in a number of countries such as Azerbaijan, China, France, Greece, Egypt, India, Iran, Israel, Italy, Mexico, Morocco, Spain and Turkey. The spice saffron is the end product of this plant. Saffron, in filaments, is the dried dark-red stigmas of *CS* flower. The weight of a single stigma is circa 2 mg and each flower has three stigmata; 150.000 flowers must be thoroughly selected separately to gain 1 kg of spice. Saffron has a characteristic color, taste and smell. From ancient to modern times the history of saffron is full of applications. It is widely utilized as a perfume, as a spice for flavoring and staining food and drink preparations. The most common way to consume saffron is still to mix it with food or to add it to any hot or warm drink [12,13].

Additionally, saffron is commonly utilized in traditional medicine, as a beneficial agent for the therapy of various pathologies of the cardiovascular, respiratory, gastrointestinal and nervous system For review see [14].

### 2.1. Chemistry of CS

The prevailing non-volatile components of the saffron are crocins, crocetin, safranal picrocrocin and flavonoids (querectin and kaempferol) [12]. The coloring components of saffron are crocins (C_44_H_64_O_24_), which are unusual water-soluble carotenoids (glycosyl esters of crocetin). The major component is a digentiobiosyl ester of crocetin (C_44_H_64_O_24_, 8,8’-diapo-*Ψ*,*Ψ*’-carotenedioic acid bis (6-0-*β*-d-glucopyranosyl-*β*-d-glucopyranosyl) ester). Safranal (C_10_H_14_O, 2,6,6-trimethyl-1,3-cyclohexadiene-1- carboxaldehyde), which is responsible for the characteristic aroma of saffron is a monoterpene aldehyde. The principal bitter-tasting substance is picrocrocin a glycoside of safranal (C_16_H_26_O_7_, 4-(*β*-d-glucopyranosyloxy)-2,6,6-trimethyl-1-cyclohexene-1- carboxaldehyde) [15,16,17].

**Figure 1:**
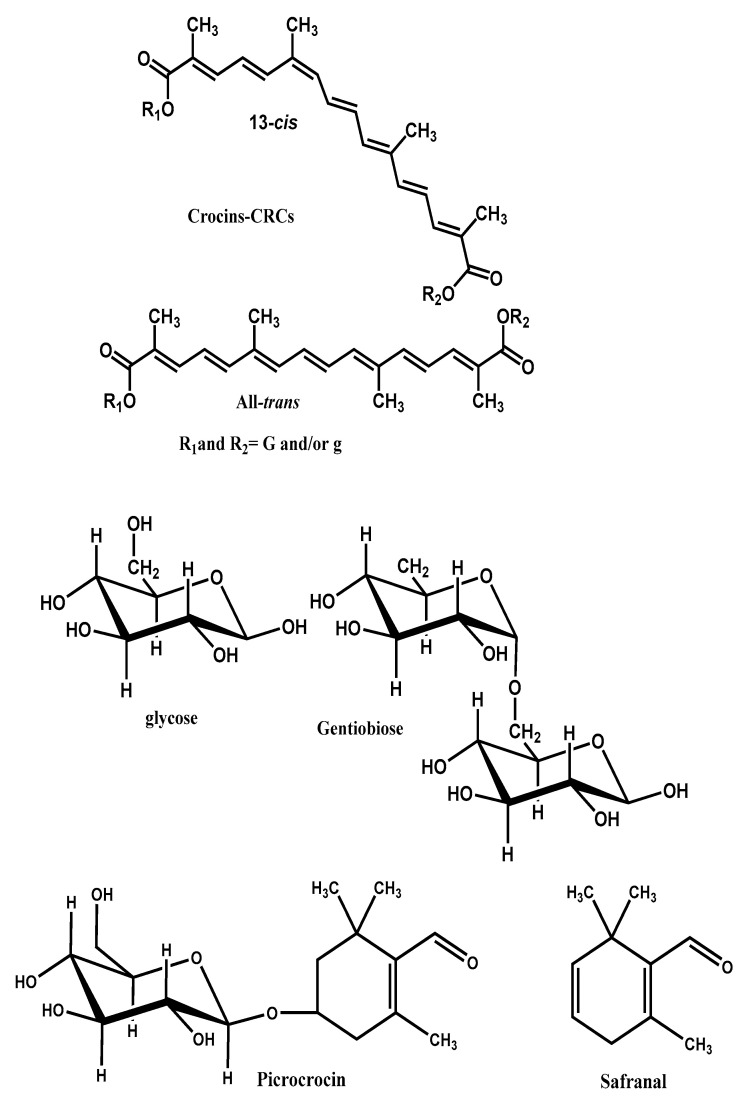
Chemical structures of saffron bioactive components. Crocins (CRCs): Glucosyl ester of Crocetin, R_1_ = â-d-Geotiobiosyl, R_2_ = â-d-Geotiobiosyl, R_1_ = â-d-Gentiobiosyl, R_2_ = â-d-glucosyl., R_1_ = â-d-Gentiobiosyl, R_2_ = H, R_1_ = â-d-glucosyl, R_2_ = â-d-glucosyl., R_1_ = â-d-glucosyl, R_2_ = H, Crocetin R_1_ = H, R_2_ = H., B. Picrocrocin., C. Safranal.

In Figure 1 the molecular structures of *CS* and its constituent crocins, picrocrocin and safranal are illustrated.

### 2.2. Pharmacology of CS and Its Bioactive Components

Based on a conspicuous number of preclinical and clinical data an exciting pharmacological profile of saffron and its bioactive ingredients is turning up.

#### 2.2.1. Effects of CS and Its Constituents on Non-Neurological/Neuropsychiatric Pathologies

In a series of preclinical in vitro and in vivo studies the anti-cancer, anti-nociceptive and anti-inflammatory properties of saffron have been revealed. Additionally, it has been reported that *CS* and its bioactive ingredients reduced atherosclerosis and hepatotoxicity, diminish hyperlipidemia, display a protective action on myocardial injury and consistently reduce blood pressure [14,18,19].

As a whole, what emerges from these preclinical findings is that *CS* and its active components appear to express a beneficial action in preclinical models of different non-neurological/neuropsychiatric pathologies. Until now, however, there is a lack of clinical results validating the therapeutic efficiency of saffron observed in the above-mentioned preclinical pathological models. Thus, clinical studies should be designed and conducted in order to properly address this important issue.

#### 2.2.2. Effects of CS and Its Constituents on Pathologies of The Central Nervous System

In a series of preclinical studies, the anticonvulsant properties of the aqueous and ethanolic extracts of *CS* and safranal have been observed and. Specifically, it has been demonstrated that *CS* extracts and safranal counteracted pentylenetetrazol-induced seizures in mice and rats and these effects seem to be mediated by their interaction with the GABAergic and opioids systems [20,21]. Further, both saffron and its active components were found to be protective in preclinical models of Parkinson’s disease (PD) and cerebral ischemia [22,23,24,25].

The efficacy of saffron and crocins to attenuate memory impairments in preclinical models associated with Alzheimer’s disease (AD), cerebral injuries, or schizophrenia is well documented For a review, see [26]. The outcome of clinical trials designed to examine the efficiency of saffron in alleviating memory problems, a common feature of AD, proposes that the effects exerted by *CS* on cognition, although modest, were similar to those displayed by the reference molecules donepezil and memantine. Importantly, in all human studies conducted, treatment with saffron, in contrast with donepezil and memantine, did not induce noticeable undesired effects [27].

Intensive preclinical research revealed a consistent antidepressant-like effect of saffron and its major constituents crocins and safranal [28]. This antidepressant-like effect of saffron observed in rodents was corroborated by clinical findings. Studies carried out in humans evidenced the efficacy of saffron in the therapy of mild-to-moderate depression [29,30]. In this context, it has been reported that saffron attenuated sexual malfunction in both males and females which was caused by the challenge with the selective serotonin re-uptake inhibitor (SSRI) antidepressant agent fluoxetine [31,32].

Up to now, few studies have been conducted aiming to investigate the potential anti-anxiety effect of *CS* and its components. In spite of the scant number of studies (preclinical and clinical), the results reported appear promising. Further research is mandatory in order to fully elucidate and establish the anxiolytic profile of saffron and its bioactive components. For review, please see [33].

#### 2.2.3. Pharmacokinetic and Safety Studies

Pharmacokinetic studies revealed that crocins, following oral administration, are not absorbed in the gastrointestinal tract (GIT) but are hydrolyzed to crocetin and in this form are absorbed in the GIT [34,35]. Crocetin is the active metabolite among which crocins exert their beneficial actions. Crocetin reaches the blood circulation and is found to be relatively quickly distributed in all tissues of the human body [36] can be partially conjugated with mono and diglucuronides in the GIT and in the liver [37]. A recent report demonstrated, however, that after oral application also crocins can be absorbed through GIT with poorer bioavailability compared to crocetin [38]. As a whole, either crocins or crocetin when applied orally, display low stability, poor absorption and low bioavailability [39]. Reportedly in this context, it has been evidenced that intraperitoneal or intravenous rather oral administration of saffron and its active components might provide higher levels of absorption and bioavailability [38,40]. In particular, Zhang and colleagues have indicated that intravenous application of crocins in rats did not reveal the presence of crocetin in plasma but solely crocins were detected, eliciting thus that hepatic metabolism of crocins would be insignificant [38]. Further studies are required, however, aiming to clarify this important issue.

Interestingly, it has been shown that crocins despite their high hydrophilic profile, similarly to crocetine [41,42], can cross the blood–brain barrier (BBB) and reach the central nervous system [40]. Concerning safranal, it can be hypothesized that might be able to penetrate the BBB since in a series of studies its anticonvulsant and antidepressant properties have been revealed [20,21,28].

Toxicological investigations performed in rodents that have received saffron extracts shown that the hematological and the biochemical parameters of the animals were not altered by treatment with saffron and remained at physiological levels [43]. In this context, it has been reported that the oral LD_50_ of *CS* was 20.7 g/kg when was delivered as a decoction in mice [44]. Further research confirmed the good safety profile of *CS* and its ingredients. Specifically, it has been observed that acute treatment of mice with saffron (up to 3 g, either orally (p.o.) or intraperitoneally (i.p.)) and repeated with crocin (15–180 mg/kg, i.p.) did not affect a series of biochemical, hematological and pathological markers recorded [45].

The outline of human studies confirmed the safe profile of *CS* extracts and crocin observed in preclinical experiments. In a double-blind, placebo-controlled study, carried out on healthy volunteers, repeated challenge with saffron (200–400 mg/day, for seven consecutive days) did not induce appreciable abnormalities. It caused only some minor consistency clinical and laboratory parameter changes such as hypotension, reduced platelets and bleeding time and increased creatinine and blood urea nitrogen levels [46].

In agreement with the above, are the findings of another clinical trial conducted on healthy participants who received 20 mg/day of crocin for 30 consecutive days. Treatment with crocin did not produce any alteration of various hematological, biochemical, hormonal and urinary parameters recorded [47]. Finally, the challenge with very high doses of saffron (1.2–2 g) in healthy volunteers caused nausea, diarrhea, vomiting, and bleeding [48]. As a whole, saffron and its main bioactive components can be considered as safe natural products displaying very low toxicity.

## 3. Effects of *CS* and Its Constituents in Schizophrenia

### 3.1. Preclinical Studies

Table 1 summarizes the existing literature regarding the effects of crocins on animal models of schizophrenia. Crocins (15–30 mg/kg, acutely) counteracted disruption of non-spatial recognition memory caused by acute administration of the NMDA receptor antagonist ketamine (3 mg/kg, acutely) in rats. This finding strongly proposes the involvement of this bioactive ingredient of *CS* in schizophrenia-related cognitive impairments. Additionally, crocins (50 mg/kg, acutely) attenuated ketamine (25 mg/kg, acutely)—induced psychotomimetic effects (hypermotility, stereotypies and ataxia) in the rat. Further, in a behavioural procedure mimicking the negative symptoms of schizophrenia (social interaction test), these active components of saffron (50 mg/kg, acutely) were found able to reduce the social isolation-induced by treatment with ketamine (8 mg/kg, sub-chronically) in rats [49].

In agreement with the above, crocins (30 mg/kg, acutely) antagonized disruption of non-spatial recognition memory caused by a single injection of the mixed DA D1/D2 receptor agonist apomorphine (1 mg/kg). By contrast, crocins failed to counteracted spatial recognition memory induced by apomorphine (1 mg/kg, acutely). It has been suggested that this dual action of crocins on recognition memory deficits observed in a dopaminergic model of amnesia might depend to differences in stimuli intensity (higher in non-spatial tasks as compared to spatial tasks) [50].

It has recently been reported that crocin attenuated schizophrenia-like symptoms in a glutamatergic model of this psychiatric disease. In particular, crocin (25 and 50 mg/kg, acutely) attenuated motor disturbances and spatial navigation impairments induced by acute administration of the NMDA receptor antagonist MK-801 (1 mg/kg) in rats [51].

It is important to emphasize that the beneficial effects of crocins, summarized in Table 1, were observed following intraperitoneal application of them in rodents. Intraperitoneal compared to oral route of administration might be of higher utility since it can be avoided the first-pass metabolism and/or gastric hydrolysis and obtain consistent bioavailability profile of the compound (elimination of liver-induced metabolism as well exposure of crocins in a low pH of the stomach [52].

### 3.2. Clinical Studies

Up to our days, clinical information dealing with a potential anti-schizophrenia efficacy of *CS* and its bioactive constituents is inconsistent. Only one clinical study was conducted aiming to evaluate the safety and the tolerability of treatment with saffron and crocin in schizophrenia patients. This was a double-blind, placebo-controlled trial and participated 61 schizophrenics. Patients were treated twice daily with saffron or crocin (15 mg) or placebo for 12 consecutive weeks. In agreement with prior reports [27,29,53], the results of this study showed that saffron extracts, safranal and crocin were well-tolerated in schizophrenics [54]. In this context, is important to emphasize that challenge with a saffron aqueous extract (30 mg/day, for 12 weeks) administered in schizophrenics on treatment with olanzapine prevented the metabolic syndrome, a well-known side effect of this atypical neuroleptic [55].

### 3.3. Potential Mechanism of Action of CS and Its Constituents in Schizophrenia

The exact mechanism(s) through which crocins exert their effects on schizophrenia-like behavior caused by glutamatergic and dopaminergic dysfunction is (are) not yet elucidated. Research is needed aiming to clarify this important issue. That schizophrenia-like effects of NMDA receptor antagonists (e.g., ketamine, MK-801) are related to increased concentrations of glutamate, hypermotility, stereotypy and cognition deficits [56,57] is well-documented. In this context, it has been reported that acute systemic administration of safranal reduced kainic acid-induced increase of extracellular glutamate concentrations in the rat hippocampus [58]. Further, it has been observed that either saffron or crocetin but not crocins partly counteract the NMDA receptor by binding to the phencyclidine (PCP) binding site of it [59]. The apparent failure of crocins to bind at the NMDA receptor might depend on pharmacokinetic issues, and in particular, their poor intestinal absorption after oral administration in rats [59]. Moreover, it has been shown that *CS* extracts and crocetin normalized excessive glutamatergic synaptic transmission in rat cortical brain slices [60]. Finally, CS extracts and crocetin were found to display a strong affinity for the sigma (σ)1 receptor [59]. As a whole, these results propose that this decrease of glutamate concentrations by *CS* and its constituents might be crucial for the beneficial action exerted by crocins on NMDA receptor antagonists-induced psychotomimetic effects and cognitive deficits.

There is poor evidence concerning the mechanism(s) by which crocins could counteract the detrimental effects of apomorphine on non-spatial recognition memory. In this context, it has been reported that apomorphine prevented the induction of long-term potentiation (LTP) which is the electrophysiological correlate of cognition [61], while crocins promote it [62].

Although the pathogenesis of schizophrenia is not yet fully clarified, a possible association with oxidative stress [5,51], inflammation [51,63] and abnormally low concentrations of different neurotrophins as is the brain-derived neurotrophic factor (BDNF) [64] has been suggested. In line with the above, in a series of reports, the pro-oxidative and pro-inflammatory profile either of NMDA receptor antagonists [51,65] or apomorphine [61] has emerged. The potent antioxidant properties of crocins may offer an alternative explanation for the beneficial effects exerted by these bioactive components of saffron in preclinical models of schizophrenia [66,67,68,69]. In this context, it has recently been demonstrated that the neuroprotective action of crocins evidenced in a preclinical glutamatergic model of schizophrenia was related to their ability to restore the expression of BDNF and that of the silent information regulator-1 (SIRT-1), a modulator of oxidative stress and inflammation, thus eliciting alleviation of the oxidative stress [51].

## 4. Conclusions

There is poor available information (either preclinical or clinical) concerning a potential beneficial role for *CS* and its bioactive constituents in the therapy of schizophrenia. In spite of it, the few preclinical data produced do not lack consistency and are really promising. The latter elicits that future research is mandatory in order to definitively establish if these compounds are suitable candidates and provide a benefit in the therapy of schizophrenia. It is important to underline the clinical efficacy expressed by saffron and its constituents in depression [28,29,30] and anxiety [33] which are typical features of patients suffering from schizophrenia [70,71] and further emphasize their good safety profile expressed in human studies.

Future research should examine the efficacy of these natural products on preclinical models resembling attentional deficits and extensively evaluate their efficacy on animal models mimicking negative symptoms of this devastating psychiatric disorder. The utilization of other than pharmacological models (e.g., neurodevelopmental, genetic etc.) will be of high value. Finally, in the case of positive preclinical findings, human studies (double-blind, placebo-controlled studies) by recruiting an appropriate number of participants should be conducted in order to evaluate the efficacy of these compounds in schizophrenia. A summary of some future research activities (either preclinical or clinical) is provided in Table 2.

## Figures and Tables

**Table 1:** Effects of crocins on preclinical models of schizophrenia.

Scheme	Agent	Dose Range	Route	Behavioral Test	Effect	Reference
Rat	Crocins	15, 30, 50 mg/kg	i.p. acute	ORT	Crocins (15, 30 mg/kg) counteracted ketamine-induced non-spatial recognition memory deficits.	[49]
	Ketamine	3 mg/kg (NORT)	i.p. acute			
	Ketamine	8 mg/kg (SI)	i.p. sub-chronic	SI	Crocins (50 mg/kg) attenuated ketamine-induced social isolation.	[49]
	Ketamine	25 mg/kg (motor activity)	i.p. acute	Motor activity, stereotypies, ataxia	Crocins (50 mg/kg) attenuated ketamine-induced hypermotility, stereotypies and ataxia.	[49]
Rat	Crocins	15, 30, 50 mg/kg	i.p. acute	ORT	Crocins (15, 30 mg/kg) counteracted ketamine-induced non-spatial recognition memory deficits.	[50]
	Apomorphine	1 mg/kg	i.p. acute			
Rat	Crocins	15, 30, 50 mg/kg	i.p. acute	OLT	No effect	[50]
	Apomorphine	1 mg/kg	i.p. acute			
Rat	Crocins	25, 50 mg/kg	i.p. acute	RR, OFT	Crocin (25, 50 mg/kg) counteracted MK-801-induced motor activity deficits.	[51]
	MK-801	1 mg/kg	i.p. acute			
	Crocins	25, 50 mg/kg	i.p. acute	MWM	Crocin (25, 50 mg/kg) counteracted MK-801-induced spatial memory deficits.	[51]
	MK-801	1 mg/kg	i.p. acute			

**Abbreviations:***i.p,* intraperitoneally; *MWM*, Morris water maze; *OFT*, open field test; *OLT*, object location task; *ORT*, object recognition task; *RR*, rotarod; *SI*, social interaction.

**Table 2:** Summary of future studies designed to evaluate the role of *Crocus sativus* and its bioactive components in schizophrenia. Key plans.

**Preclinical research**
Acute vs. repeated drug treatment Not pharmacological animal models of schizophrenia (genetic, neonatal ventral hippocampal lesions models etc) Evaluation of the effects of saffron and its constituents in animal models of attentional deficits Further evaluation of the effects of saffron and its constituents in animal models resembling cognitive impairments and negative symptoms of schizophrenia Investigation of potential mechanism(s) of action underlying the beneficial effects of saffron and its constituents observed in preclinical studies (molecular, biochemical, neurochemical, electrophysiological studies etc)
**Clinical research**
Multi-center, double-blind, placebo-controlled studies Studies of the effects of saffron alone in schizophrenia patients Studies of the effects of saffron in combination with atypical antipsychotics in schizophrenia patients Use of broad dose range of *C. sativus* Appropriate number of participants

## Data Availability

The data presented in this study are available on request from the corresponding author.
